# The effects of the combined use of carbon quantum dots and antibacterial agents on pathogenic bacteria

**DOI:** 10.55730/1300-0152.2774

**Published:** 2025-09-04

**Authors:** Derya DOĞANAY, İbrahim Serkan AVŞAR, Şevval Maral ÖZCAN AYKOL, Gamze ÇAMLIK, Besa BİLAKAYA, İsmail Tuncer DEĞİM

**Affiliations:** 1 Department of Pharmaceutical Microbiology, Hamidiye Faculty of Pharmacy, University of Health Science, İstanbul, Turkiye; 2 Department of Pharmacology, Faculty of Pharmacy, Tokat Gaziosmanpasa University, Tokat, Turkiye; 3 Department of Pharmaceutical Microbiology, Faculty of Pharmacy, Biruni University, İstanbul, Turkiye; 4 Department of Pharmaceutical Technology, Faculty of Pharmacy, Biruni University, İstanbul, Turkiye

**Keywords:** Composite carbon quantum dots, quantum dots, antibiotic resistance, antibacterial activity, teicoplanin, synergism

## Abstract

**Background/aim:**

This study evaluates the challenges associated with overcoming antimicrobial resistance and innovative approaches to combat multidrug-resistant (MDR) bacterial infections.

**Materials and methods:**

Novel codoped carbon quantum dots (CCQDs) were synthesized using citric acid as the carbon source and L-cysteine as the nitrogen codoping atom. The formulation in which citric acid was retained was designated as CCQDs-1, whereas the purified version, from which citric acid was removed, was termed CCQDs-2. The antibacterial properties of CCQDs-1 and CCQDs-2 were compared using the agar well diffusion method. This study comprehensively characterizes these nanomaterials and evaluates their antibacterial potential, both alone and in combination with antibiotics, against a spectrum of gram-positive (G+) and gram-negative (G−) bacterial strains.

**Results:**

The study demonstrates the significant antibacterial efficacy of CCQDs, with notable variations observed between citric acid-containing and citric acid-neutralized formulations. The QDs exhibited remarkable characteristics, including a quantum yield of 90.3%–90.6%, intense fluorescence, and distinctive interactions with various antibiotics. In addition to their intrinsic antibacterial activity, the QDs also exhibited synergistic effects when combined with certain antibiotics. A synergistic effect was particularly observed when CCQDs-2 were combined with antibiotics such as gentamicin, levofloxacin, and clindamycin, suggesting potential mechanisms such as membrane permeability disruption and efflux pump saturation.

**Conclusion:**

These findings underscore the promising potential of carbon-based QDs as innovative, biocompatible solutions to address the critical global challenge of antimicrobial resistance.

## 1. Introduction

The increase in bacterial infections and drug resistance against antibacterial agents has emerged as a critical global health problem. The mortality and morbidity caused by conventional treatments have made the development of alternative agents against infections an urgent necessity (Sugden et al., 2016). While many traditional antibacterial drugs fail to overcome adaptive bacterial resistance, a sophisticated approach is required to address this dynamic challenge.

Traditional antimicrobials encounter difficulties in penetrating gram-negative (G−) pathogen cell walls; however, nanoparticle-based therapeutic agents can easily cross cell walls with considerable stability due to their small size (Loo et al., 2005; Soo Choi et al., 2007; Yong et al., 2013; Courtney et al., 2016a, 2017b). The development of new nanomaterials remains a primary objective in the field to generate innovative treatment methods or alternatives for controlling MDR bacteria-based infections (Huh and Kwon, 2011).

Quantum dots (QDs) are novel fluorescent nanoparticles developed in recent years. Compared to traditional materials, QDs possess unique physical and chemical properties, including high stability, good emission, and high quantum yield. (Jahangir et al., 2019; Wang et al., 2019). QDs functionalized with polymers exhibit more promising properties, and they may have enhanced antibacterial activities (Coleman and Moffitt, 2018; Owusu et al., 2019). Currently, functionalized QDs are gaining attention due to their distinctive antibacterial mechanisms, and it is hypothesized that they may serve as an effective alternative to traditional antibacterial drugs.

QDs show antibacterial effects by breaking down the cell wall/cell membrane of microbial organisms, killing bacterial cells with reactive oxygen species (ROS), or binding to nuclear material and inhibiting cell proliferation (Herman and Herman, 2014; Ivask et al., 2014).

The antibacterial properties of nanoparticles have attracted considerable attention; however, their high cytotoxicity, particularly in the case of metal and metal oxide nanoparticles, remains a significant barrier to clinical application, underscoring toxicity as a critical concern in the development of new agents against multidrug-resistant (MDR) bacterial strains (Chen et al., 2014; Ivask et al., 2014). Due to their nature (such as nontoxicity, abundance, and low cost), carbon QDs (CQDs) do not possess this disadvantage in the field and render them competent for developing drug delivery systems (Bourlinos et al., 2008; Wang et al., 2013). Moreover, their biocompatibility and ability to selectively generate ROS, such as superoxide, contribute to their antimicrobial efficacy. In vitro measurements have demonstrated that low nanomolar doses of superoxide can kill various MDR pathogens without affecting the growth or vital characteristics of the mammalian host cell (Courtney et al., 2016; Courtney et al., 2017; Goodman et al., 2018; Levy et al., 2018).

In this study, a new generation of CCQDs, along with their purified counterparts, was synthesized, characterized, and evaluated for their antimicrobial potential. Gram-positive (G+) bacteria and G− bacteria were employed to test and compare the antimicrobial effects of CCQDs in the presence and absence of conventional antibacterial drugs.

## 2. Materials and methods

### 2.1. Synthesis of CCQDs

Citric acid monohydrate (CAMH), boric acid (BA), other chemicals, and solvents used in the QD synthesis were all analytical grade. In addition, a dialysis membrane (molecular weight cut-off value 12 kDa) was used.

A novel QD formulation was developed and subsequently divided into two forms: unpurified and purified. The purification process involved the neutralization of citric acid and adjustment of the pH to eliminate its residual effects. CAMH (0.1 g) and L-cysteine (0.005 g) were dissolved in 2 mL of distilled water. The same solution was subjected to microwave synthesis and then dialyzed (and coded as CCQDs-1).

A 5 mL aliquot of CCQDs-1 solution was taken, and 1 M NaOH was added to it. An excess amount of absolute alcohol was then added, and all solvents were evaporated at room temperature using a rotary evaporator. The solution subsequently was centrifuged at 4500 rpm. All crystals (such as citrate and borate) were removed. After evaporation of the alcohol in the filtrate, the QDs were redispersed in water, and the volume was adjusted to the original value. This purified formulation was coded as CCQDs-2.

The program for the microwave synthesis was as follows:

1. Initial heating: 0–140 °C for 5 min
2. Holding: 140 °C for 20 min
3. Cooling: 140–60 °C, with no time limitation
4. Stirring rate for all steps: 500 rpm

### 2.2. Characterization of CCQDs

#### 2.2.1. Physical appearance

The prepared CCQDs were examined using transmission electron microscopy (TEM; Carl Zeiss EM 900) after subsequent dilutions with ultrapure water.

#### 2.2.2. Particle size and zeta potential

The CCQDs were characterized by their particle sizes (PSs), size distributions, and zeta potentials (ZPs) using a Litesizer 500 (Anton Paar).

#### 2.2.3. Fluorescence and quantum yield

The fluorescent properties of the CCQDs were determined under ultraviolet (UV) light. An accurate method was employed to determine the quantum yields. A series of solutions with optical densities ranging from 0.01 to 0.1 was prepared, and fluorescence intensity was plotted against optical density. The slope of the line was calculated. Quantum yield was calculated using the following equation (Coleman and Moffitt, 2018):


$$Q=Q_{R}\left(\frac{Grad}{Grad_{R}}\right)\;\left(\frac{n^{2}}{n_{R}^{2}}\right)$$

Q and QR are the quantum yields, and n is the refractive index of the solution (n = 1.3325 for test (water) and 1.3333 for reference (quinine sulfate in 0.1 M H_2_SO_4_)).

### 2.3. Antibacterial activity

The antimicrobial activities of the CCQDs (CCQDs-1 and CCQDs-2) and their combinations with antibiotics (1:1) were determined. Antimicrobial activity against G (+) bacteria, i.e., *Staphylococcus haemolyticus* American Type Culture Collection (ATCC) 43252, *S. aureus* ATCC 25923, and methicillin-resistant *S. aureus* (MRSA) ATCC 43300; and G (−) bacteria, i.e., *Acinetobacter baumannii* ATCC 19606, *Pseudomonas aeruginosa* ATCC 27853, and *Klebsiella pneumoniae* ATCC 700603, was determined using the well diffusion method.

The 24-h fresh cultures of all bacterial strains were prepared in Mueller–Hinton broth (MHB), and the bacterial concentration was adjusted to 0.5 McFarland (1.5 × 10^8^ colony-forming units [cfu]/mL). Mueller–Hinton agar (MHA) media were prepared, and 6-mm diameter wells were created using sterile glass Pasteur pipettes. A total of 100 μL of the bacterial cultures was transferred to the MHA and inoculated using the spread plate method. A total of 30 μL of CCQDs or CCQDs plus antibiotic mixture was added to the wells. Plates were incubated aerobically for 24 h at 37 °C. Following incubation, inhibition zone diameters (mm) were measured using a caliper (Clinical and Laboratory Standards Institute [CLSI], 2022).

A group of antibiotics was used, including ampicillin (10 μg), gentamicin (10 μg), teicoplanin (30 μg), clindamycin (2 μg), levofloxacin (5 μg), and cefazolin (30 μg), to evaluate their potential synergistic effects with the synthesized QDs. All experiments were performed in triplicate, and the results were assessed using the average values.

The synergistic effects of CCQDs in combination with antibiotics were calculated using a custom-designed web application developed in accordance with the mathematical formula published by Arsène (2021).

## 3. Results and discussion

Antimicrobial drug resistance represents one of the principal causes of mortality on a global scale. The development of novel alternatives against MDR bacteria has become an urgent imperative. Otherwise, public health challenges stemming from resistant bacterial strains will proliferate worldwide.

**Figure 1:**
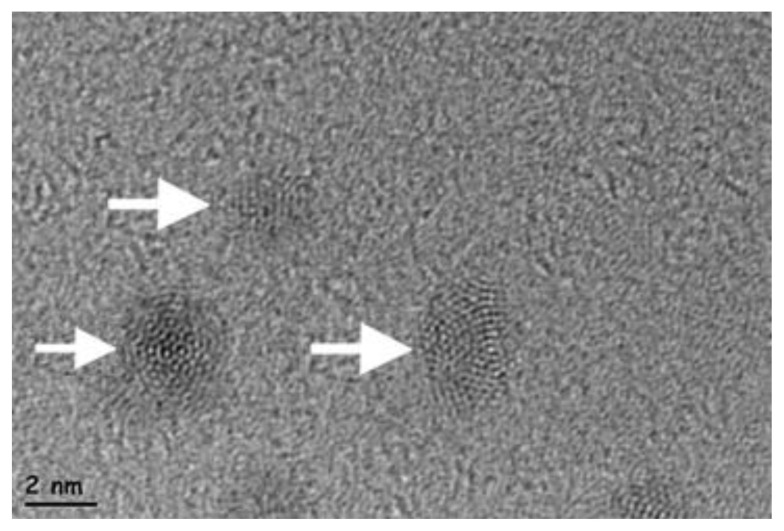
TEM images showing the physical morphology of CCQDs.

In this study, novel CCQDs were synthesized and comprehensively characterized through chemical analysis. Upon UV light examination, these QDs demonstrated a nuanced emission color mechanism influenced not merely by particle size, but significantly by surface roughness and functionalization. Nitrogen doping via L-cysteine facilitated specific atomic orientation, resulting in a remarkably bright blue luminescence. The zeta potential and polydispersity index (PDI) values substantiated the CCQDs’ remarkable stability and homogeneity, providing critical insights into their structural integrity (Table 1, Figure 1).

**Figure 2:**
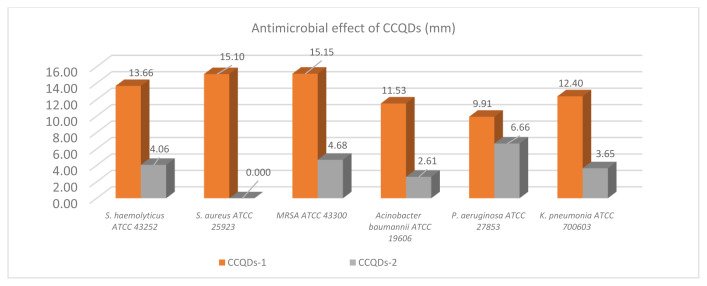
Antimicrobial activity of CCQDs.

The QDs exhibited diminutive particle dimensions, thereby enabling enhanced penetrative capabilities. The quantum yields were 90.3% and 90.6%, respectively, and the synthesis technique unequivocally demonstrated exceptional performance. These novel compounds were tested against bacterial strains. They demonstrated significant antibacterial activities to such an extent that they exhibited higher or comparable antibacterial activities compared with conventional antibacterial drugs. These observations were valid for both G (−) and G (+) bacteria. Therefore, our novel CCQDs have the potential to be utilized against both G (−) and G (+) bacterial strains (Figure 2).

In the study, the CCQDs exhibited significant variability in their antibacterial properties, depending on citric acid concentration. (Figure 2).

Our study results demonstrate that CCQDs-1 exhibit higher antibacterial efficacy in the presence of citric acid. However, upon citric acid neutralization and purification of CCQDs-2, a notable reduction in antibacterial activity was observed. This finding suggests that in the absence of citric acid, the QDs’ genuine antibacterial effects can be better understood, as CCQDs-2, despite showing lower efficacy compared with CCQDs-1, is more significant due to its purity and lack of citric acid. Furthermore, while CCQDs-1 containing citric acid demonstrated antibacterial effects on both G (+) and G (−) bacteria, CCQDs-2 purified by neutralizing citric acid showed no effect on *S*. *aureus* ATCC 25923. When CCQDs-1 were comprehensively evaluated, they proved more effective against G (+) bacteria, whereas CCQDs-2 demonstrated greater efficacy against G (−) bacteria. The difference in effect can be attributed to the unique cell wall structure of G (−) bacteria, with pH adjustment potentially enhancing the penetration of QDs through the bacterial membrane (Figure 2). Furthermore, our findings indicate that if the CQDs obtained after synthesis, similar to those in our study, are purified solely via membrane-assisted dialysis without removing citric acid by means of a neutralizing agent such as NaOH and without adjusting the pH, the inherent effects of the CQDs will be overshadowed by the antibacterial properties of citric acid. Nevertheless, we have observed that numerous studies in the literature report findings obtained without any pH adjustment.

In the combination of CCQDs-1 with antibacterial agents, the limited synergistic effect or even the emergence of antagonism can be associated with citric acid content. However, combinations of citric acid-free CCQDs-2 demonstrated a notable additive and synergistic effect on different bacterial strains (Tables 2 and 3).

**Figure 3:**
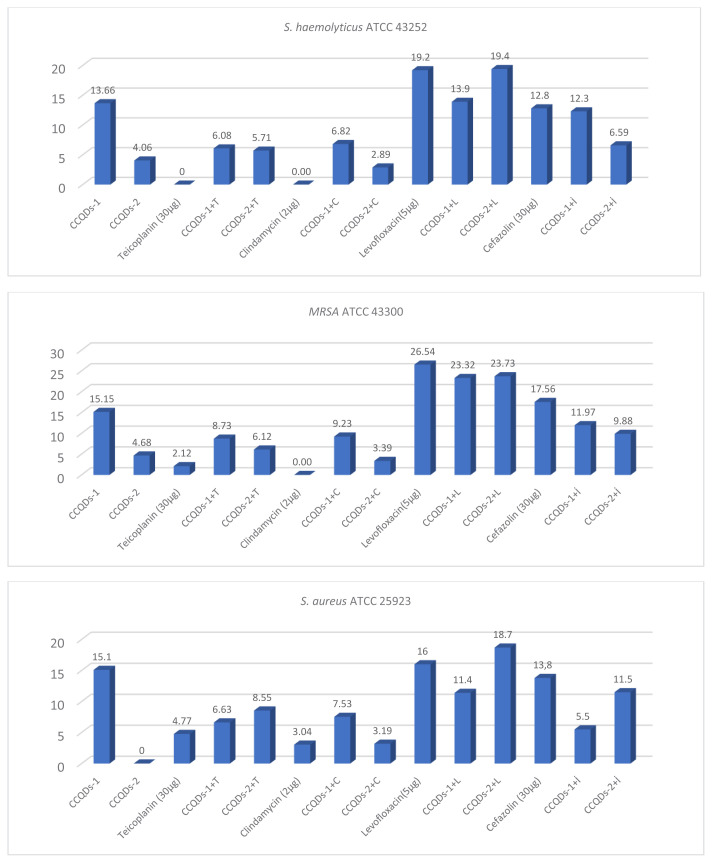
Antimicrobial effect of antibiotics and CCQDs on gram (+) bacteria (mm).

*Staphylococcus haemolyticus*, *S. aureus*, and MRSA bacterial strains were found to have developed resistance to narrow-spectrum Teicoplanin (resistant ≤10 mm) and Clindamycin (resistant ≤14 mm) antibiotics (Figure 3). When Teicoplanin was combined with CCQDs-2 in a 1:1 ratio against G (+) bacteria at resistance limits, a potential synergistic effect emerged.

All G (+) bacterial strains used in the study were determined to be susceptible or intermediately susceptible to Levofloxacin (susceptible ≥19 mm, intermediate = 16–18 mm). The MRSA strain showed intermediate susceptibility to Cefazolin (intermediate = 15–17 mm; resistant ≤14 mm), while other bacteria were resistant.

When Cefazolin was individually combined with CCQDs-1 and CCQDs-2 in 1:1 ratios against G (+) bacteria, categorical antagonism was observed between Cefazolin and CCQDs-1, and an antagonistic effect was noted with CCQDs-2.

In the study, Levofloxacin, which was susceptible to *S. haemolyticus* (ATCC 43252) and *S. aureus* (ATCC 25923), demonstrated a synergistic effect when combined with CCQDs-2 in a 1:1 ratio. Conversely, an antagonistic effect was observed on the MRSA (ATCC 43300) bacterial strains (Table 2, Figure 3).

**Figure 4:**
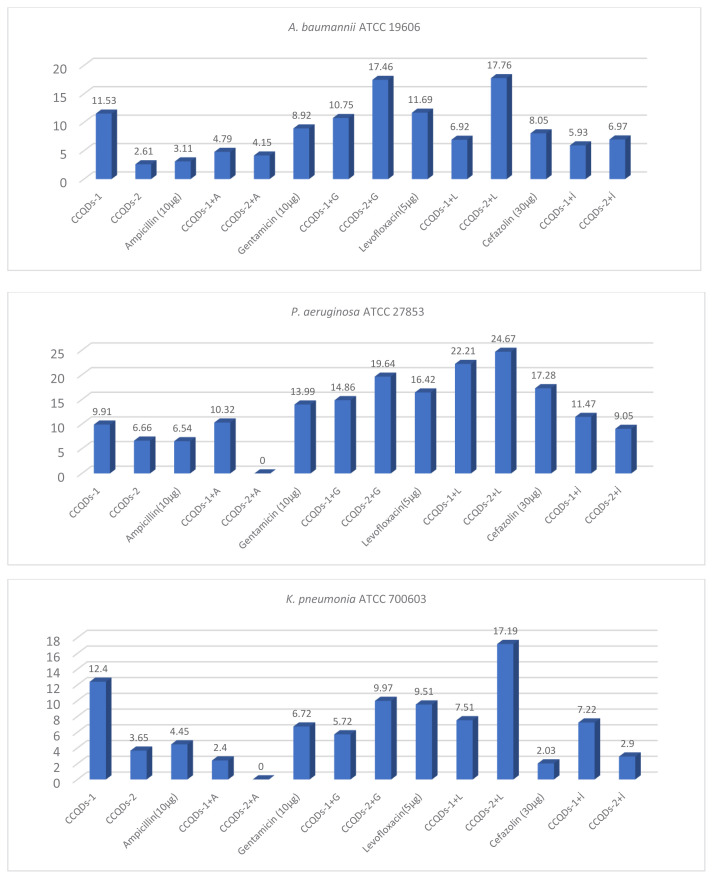
Antimicrobial effect of antibiotics and CCQDs on gram (−) bacteria (mm).

Against all tested G (−) bacterial strains, CCQDs-2, when combined, increased the effectiveness of gentamicin and levofloxacin, even bringing them to susceptible levels. The combination of CCQDs-2 with these antibiotics demonstrated potential synergistic effects on the G (−) bacteria used in the study (Figure 4). When ampicillin was combined with CCQDs-2, it completely lost its effectiveness against *K. pneumoniae* ATCC 700603 and *P. aeruginosa*-ATCC 27853 bacteria, with categorical antagonism determined between them. The combination of cefazolin with CCQDs-2 showed antagonistic effects on all G (−) bacteria (Table 3). While Ampicillin and Cefazolin are known to exhibit antibacterial activity by disrupting bacterial cell wall synthesis and activating autolytic enzymes, gentamicin inhibits protein synthesis by binding to the 30S subunits of ribosomes, and Levofloxacin acts on deoxyribonucleic acid (DNA) gyrase complex and topoisomerase IV. The differences in the mechanism of action or bacterial resistance mechanisms to these agents could explain the varied effects observed.

Clindamycin, gentamicin, and levofloxacin, which exert their effects by binding to intracellular targets, demonstrate synergism when combined with CCQDs. This can be explained by the increased penetration of these agents into the cell due to the cell membrane and cell wall permeability disruption by CCQDs. Additionally, one of the mechanisms of antibacterial resistance against these agents is the efflux of antibacterial agents from the cell through efflux pumps. However, CCQDs may prevent the extrusion of the antibacterial agent by playing an alternative substrate role that saturates efflux pumps. Moreover, CCQDs can enhance the efficacy of these antibacterial agents through their effects on bacterial ribosomes, nucleic acids, and proteins.

Our results are in line with the study by Thakur et al. (2014), which demonstrated that conjugating ciprofloxacin, a fluoroquinolone antibiotic, with CQDs enhanced its antimicrobial activity. In our study, a similar synergistic effect was observed between CCQDs and levofloxacin, another member of the fluoroquinolone class with an identical mechanism of action. These parallel findings suggest that CQDs may enhance the intracellular accumulation or activity of fluoroquinolones, potentially by increasing membrane permeability or interfering with efflux mechanisms.

Teicoplanin, ampicillin, and cefazolin, which act by disrupting cell wall synthesis, exhibited different combination profiles. This can be explained by Teicoplanin’s more substantial molecular structure. CCQDs may tend to interact more extensively with agents possessing higher molecular weight (MW). In a study by Yang et al. (2018), QDs with carboxyl and amino modifications were used to understand the effects of MW fractionated humic acid (Mf-HA) on the transport and retention behavior of nanoparticles. In this study, Mf-HAs were found to have significant effects on the transport and retention behavior of QDs, which was highly dependent on the MW of Mf-HA. In addition, the surface coating and cation types of QDs were also shown to have significant effects on the transport behavior of QDs. In another study, Liu et al. (2024) investigated the effect of lignin molecular weight on the properties of L-CQDs. By sequentially fractionating five lignin samples with molecular weights ranging from 7100 to 2000 Da, they demonstrated that lower molecular weight units facilitate the formation of highly conjugated carbon core structures, thereby enhancing the fluorescence performance of the resulting L-CQDs. Studies have shown that the behavior of prepared QDs significantly depends on their shapes, and because of these surface characteristics, including sizes and MW, interactions of substances with QDs having different molecular weights and structural or surface properties become important parameters. In particular, molecules with different MW and structural properties appear to play a decisive role in the transport and optical properties of QDs. CCQDs tend to interact more with agents possessing higher MW because molecules with high MW are as hydrophobic as CCQDs. This striking observation will be one of the primary research points in our future studies to test whether CCQDs directly interact with antibacterial drugs. Another potential explanation is that CCQDs and antibacterial drugs might neutralize each other’s mechanisms of action. These aspects will be comprehensively investigated in our future research, focusing on determining the mechanisms of action of these CCQDs against G (−) and G (+) bacteria. In our subsequent studies, we will also concentrate on evaluating the in vivo efficacy of these CCQDs against infections.

## 4. Conclusion

In conclusion, our study represents the first investigation to evaluate the antibiotic-enhancing potential of QDs across multiple classes of antibiotics and a diverse range of bacterial strains. This comprehensive investigation of CCQDs reveals their promising potential as innovative antibacterial agents against MDR bacterial strains, with antibacterial efficacy varying significantly depending on citric acid concentration and QD derivatives.

The study’s exploration of antibiotic combinations with CCQDs unveiled intriguing synergistic and antagonistic interactions, suggesting that these QDs could potentially modulate antibacterial agents’ effectiveness through mechanisms such as membrane permeability disruption, efflux pump saturation, and interference with cellular processes.

While our research provides valuable insights, it also underscores the need for further investigations.

These CQDs represent a promising avenue in addressing the global challenge of antimicrobial resistance, offering potentially biocompatible and effective solutions against challenging bacterial infections.

## Figures and Tables

**Table 1:** Particle size, zeta potential, and polydispersity index of prepared CCDQs.

CCQDs	Particle size (nm)	Zeta potential (mV)	Polydispersity index (%) (PDI %)
CCQDs-1	0.98 ± 0.04	10.04 ± 0.25	16.13 ± 0.31
CCQDs-2	1.02 ± 0.15	15.14 ± 0.16	15.07 ± 0.11

**Table 2:** Synergistic effect of antibiotic CCQDs on gram (+) bacteria.

Effect of Q dots on antibiotic activity							

Gram (+) bacteria							

*S. haemolyticus* ATCC 43252							

Antibiotic	Susceptibility interpretation	*CCQDs-1 Antibiotic value/combination effec*t			*CCQDs-2 + Antibiotic value/combination effect*		
Teicoplanin (30 μg)	R	(−)	59.25	Antagonism	(+)	56.51	Potential synergistic effect or additive effect
Clindamycin (2 μg)	R	(−)	66.70	Antagonism	(+)	27.61	Antagonism
Levofloxacin (5 μg)	S	(−)	−0.26	Antagonism	(+)	3.79	Potential synergistic effect or additive effect
Cefazolin (30 μg)	R	(−)	−0.14	Categorical antagonism	(−)	0.14	Antagonism

***S. aureus*** ** ATCC 25923**							

Teicoplanin (30 μg)	R	(−)	−0.17	Antagonism	(+)	85.29	Potential synergistic effect or additive effect
Clindamycin (2 μg)	R	(−)	0.98	Antagonism	(+)	30.95	Potential synergistic effect or additive effect
Levofloxacin (5 μg)	I	()	−0.53	Categorical antagonism	(+)	186.17	Potential synergistic effect or additive effect
Cefazolin (30) μg)	R	(−)	−1.24	Categorical antagonism	(+)	113.83	Antagonism

**MRSA ATCC 43300**							

Teicoplanin (30 μg)	R	(+)	2.69	Antagonism	(+)	2.19	Potential synergistic effect or additive effect
Clindamycin (2) μg)	R	(+)	90.91	Antagonism	(+)	32.62	Antagonism
Levofloxacin (5μg)	S	(+)	0.42	Antagonism	(+)	3.96	Antagonism
Cefazolin (30) μg)	I	(−)	−0.53	Categorical antagonism	(−)	0.67	Antagonism

R: Resistant

S: Sensitive

I: Intermediate (sensitive at higher dose)

(+) Value is above the average of Q dot and ANTIBIOTIC

(−) Value is below the average of Q dot and ANTIBIOTIC

Antagonism: A substance exhibits an antagonistic effect

Categorical antagonism: Both substances are antagonistic

**Table 3:** Synergistic effect of antibiotic CCQDs on gram (−) bacteria.

Effect of Q dots on antibiotic activity							

Gram (−) bacteria							

*A. baumannii* ATCC 19606							

Antibiotic	Susceptibility interpretation	*CCQDs-1 Antibiotic value/combination effect*			*CCQDs-2 + Antibiotic value/combination effect*		
Teicoplanin (30 μg)	R	(−)	−0.04	Antagonism	(+)	0.92	Potential synergistic effect or additive effect
Clindamycin (2 μg)	R	(+)	0.14	Antagonism	(+)	6.65	Potential synergistic effect or additive effect
Levofloxacin (5 μg)	R	(−)	−0.81	Categorical antagonism	(+)	6.32	Potential synergistic effect or additive effect
Cefazolin (30 μg)	No Data	(−)	−0.75	Categorical antagonism	(+)	1.54	Antagonism

***P. aeruginosa*** ** ATCC 27853**							

Teicoplanin (30 μg)	R	(+)	0.62	Potential synergistic effect or additive effect	(−)	−1.97	Categorical antagonism
Clindamycin (2 μg)	I	(+)	0.56	Potential synergistic effect or additive effect	(+)	2.35	Potential synergistic effect or additive effect
Levofloxacin (5 μg)	I	(+)	1.59	Potential synergistic effect or additive effect	(+)	3.21	Potential synergistic effect or additive effect
Cefazolin (30 μg)	No Data	(−)	−0.18	Antagonism	(−)	−0.56	Antagonism

***K. pneumoniae*** ** ATCC 700603**							

Teicoplanin (30 μg)	R	(−)	−1.27	Categorical antagonism	(−)	−1.95	Categorical antagonism
Clindamycin (2 μg)	R	(−)	−0.69	Categorical antagonism	(+)	2.22	Potential synergistic effect or additive effect
Levofloxacin (5 μg)	R	(−)	−0.60	Categorical antagonism	(+)	4.52	Potential synergistic effect or additive effect
Cefazolin (30 μg)	R	(+)	2.14	Antagonism	(−)	0.22	Antagonism

R: Resistant

S: Sensitive

I: Intermediate (sensitive at higher dose)

(+) Value is above the average of Q dot and ANTIBIOTIC

(−) Value is below the average of Q dot and ANTIBIOTIC

Antagonism: a substance exhibits an antagonistic effect

Categorical antagonism: both substances are antagonistic
